# Study on the performance of open countercurrent heat source tower under low temperature and high humidity environment

**DOI:** 10.1038/s41598-025-09378-0

**Published:** 2025-07-17

**Authors:** Shuangying Yang, Peng Liu, Dinggao Xiao, Yuan Li, Yaxin Yang, Yongfa Wang

**Affiliations:** 1 School of Civil Engineering, Guizhou University, Guiyang, 550025 China; 2 Guiyang Architectural Design and Surveying Prospecting Co., Ltd., Guiyang, 550082 China; 3 Guizhou CSCEC Architectural Research and Design Institute Co., Ltd., Guiyang, 550006 China

**Keywords:** Low-temperature and high-humidity, Heat and mass transfer, Energy-saving, Performance coefficient, Numerical simulation

## Abstract

This study aims to investigate the efficient operation characteristics and energy-saving effects of the heat source tower heat pump system in low temperature and high humidity environments. First, the heat and mass transfer model of an open countercurrent heat source tower was established and the heat and mass transfer performance of the heat source tower was investigated. It was obtained that the inlet and outlet solution temperatures had a significant effect on the sensible heat exchange, and the relative humidity also had a great influence on the latent heat exchange, while the inlet solution temperature was negatively correlated with the amount of heat transfer. Finally, using actual engineering cases, the heating efficiency of the heat source tower is determined. In the low temperature and high humidity environment of January, the coefficient of performance of the heat source tower ranges from 2.18 to 2.94. Throughout the entire heating season, the seasonal performance coefficient of the heat source tower is 3.18. The research findings of this study provide a theoretical basis for the application of the heat source tower in low temperature and high humidity environments.

## Introduction

### Introduction to the topic

In China, approximately 25% of the total energy consumption is attributed to buildings, and air conditioning systems alone contribute nearly 40% to the energy usage in buildings^1^. Consequently, there is a growing inclination towards reducing building energy consumption and developing more efficient air conditioning systems is of significant importance in this approach^2^. Furthermore, with the proposal of the dual carbon goals^3^, energy-saving upgrades for air conditioning and heating systems are a crucial step in reducing building energy consumption.

Currently, the air-source heat pump^4^ is the most widely used air conditioning system due to its energy efficiency and eco-friendly nature, encompassing both cooling and heating functionalities. However, air source heat pumps are still challenged by the frost problem in winter^5^, and the heating efficiency and capacity are significantly reduced during frost^6^. There are many existing defrosting and frost suppression methods, but these defrosting or frost suppression methods add additional energy consumption^7–10^. Ground source heat pumps, known for their clean and sustainable cooling and heating capabilities^11^, are inherently exempt from frosting issues. However, their application is constrained by geographical and geological conditions, along with challenges such as soil heat imbalance and substantial initial investment^12,13^. In contrast, the heat source tower (HST) heat pump is an innovative cold and heat source system with no geographical or geological limitations that is free from frosting concerns. Moreover, its initial investment cost is relatively reasonable^14^, indicating promising prospects for development.

The HST heat pump technology efficiently harnesses low-grade heat energy from cold and humid air by utilizing a carrier medium with a freezing point below 0 °C during winter, achieved through the interaction of air and water. By supplying a minimal amount of high-quality energy to the HST heat pump unit, low-quality heat energy from the low-temperature environment can be transferred to high-quality heat energy for heating and cooling buildings. HST systems are categorized into open and closed systems based on the direct contact between the circulating medium and the air. Additionally, they can be classified as countercurrent or crosscurrent systems based on the flow direction of the circulating medium and air. Open HST systems feature a simple structure, low cost, and high heat exchange efficiency. However, they are prone to antifreeze solution drift, and their freezing point temperature is unstable. Closed HST systems, on the other hand, prevent antifreeze solution drift, offering stable freezing temperatures, but they come with higher costs and lower heat exchange efficiency. The difference between countercurrent and crosscurrent HST systems lies primarily in their structure, size, and water spray density. In countercurrent systems, air passes through the packing from the bottom up, whereas in crosscurrent systems, air moves through the packing horizontally. Countercurrent HST systems exhibit high heat transfer efficiency, while crosscurrent systems typically have a smaller volume.

In the southern Guizhou region of China, with low temperature and high humidity in winter (outdoor air temperature ≤ 5 °C and relative humidity ≥ 70%)^15^, the outdoor ambient air contains a large amount of low-grade heat energy, which can provide heat source for heat pump units and belongs to renewable energy. The heat pump system, based on the original chiller, eliminates auxiliary heat sources such as boilers and absorbs low-grade heat energy in the air through the HST in winter, realizing the triple supply of cooling, heating, air conditioning and domestic hot water.

### Review of research papers

Currently, the research on HSTs mainly focuses on the heat mass transfer law in the tower and the performance of the heat pump system. Fujita et al.^16,17^ constructed a heat transfer model for an HST using enthalpy as the driving potential difference for the total heat transfer and solved for the volumetric bulk mass coefficient driven by the enthalpy difference. Song et al.^18^ experimentally investigated a crossflow HST and constructed a correlation equation for the heat mass transfer coefficient. Based on Song et al.^18^ study, Zendehboudi et al.^19^ applied the improved non-dominated genetic algorithm NSGA II to optimize the input parameter selection of the neural network, deeply analyzed the performance characteristics of the crossflow closed-HST, and established the corresponding neural network model. Huang et al.^20^ performed numerical simulations and modeling for an open crossflow HST in a low-temperature environment, revealing the important influence of latent heat. Huang et al.^21^ on the other hand studied an open crossflow HST with ethylene glycol as antifreeze and developed a coupled model and correlation equation for heat and mass transfer. In addition, in a study of open countercurrent HSTs, Tan et al.^22^ proposed a method for evaluating the heat and mass transfer characteristics of countercurrent HSTs and verified their accuracy through field tests. On this basis, Zhang et al.^23^ established a coupled transfer model to study the thermal behavior under different working conditions. Cui et al.^24,25^ found that upward/downward spraying could enhance efficiency. Lu et al.^26^ proposed a mathematical model with a variable Lewis number to investigate the effect of the operating environment on the thermal performance of the tower. In order to study the energy consumption and system efficiency of HST heat pumps, Zhao et al.^1^ studied HST heat pumps in low humidity areas, proposed a new multi-mode HST heat pump and obtained data such as its cooling and heating efficiency throughout the year. Xiao et al.^27^ proposed an HST heat pump with a self-accumulator and investigated the operating characteristics of the system in three heating modes to obtain parameters such as heating capacity and efficiency. Han et al.^28^ tested a freeze-concentration-based solution regeneration device in an HST heat pump system using an enthalpy difference laboratory to evaluate its performance in terms of solution regeneration and system cooling energy efficiency.

According to the literature review, current research on HSTs is lacking in low-temperature and high-humidity environments, and the long-term comprehensive energy efficiency of heat pump systems in HSTs in real projects needs further study. In one of our previous studies^15^, we proposed a mathematical model of an open countercurrent HST, solved the model by finite difference method, predicted the exit air enthalpy and moisture content, enthalpy, and the mass flow rate of the exit solution, parametrically investigated the effects of operational and environmental parameters on heat and mass transfer in the HST based on the model with the three evaluating indexes of latent heat ratio, tower effectiveness, and thermal efficiency; and compared the energy efficiency and economy of HST heat pumps and air-source heat pumps by using the coefficient of performance. However, current energy efficiency studies on HST heat pump systems lack comprehensive seasonal evaluation throughout the entire heating season. To better assess their integrated energy performance under low-temperature, high-humidity conditions, research on the system’s overall energy efficiency across the full heating season is required. In addition, it is necessary to explore the influence of air and solution temperatures on the HST due to the large influence of air and solution temperatures on the HST; therefore, this paper improves the model on the basis of previous mathematical models and investigates the influence law of air and solution temperatures on the heat and mass transfer in the HST.

This study aims to establish a heat and mass transfer model for open countercurrent HSTs and validate the model’s accuracy using measured data. Subsequently, based on this model, we investigate the heat and mass transfer performance of HSTs under low-temperature and high-humidity conditions. Finally, the Coefficient of Performance (*COP*) and Seasonal Coefficient of Performance for Heating (*SCOP*) are adopted to evaluate the heating performance of the heat pump system. Through long-term field testing of an actual engineering case, the integrated energy efficiency of the HST heat pump system during the heating season was determined. This work provides a theoretical foundation for applying open-type counterflow HSTs in low-temperature, high-humidity environments.

## Mathematical model of HST

### Working principle of HST

**Fig. 1:**
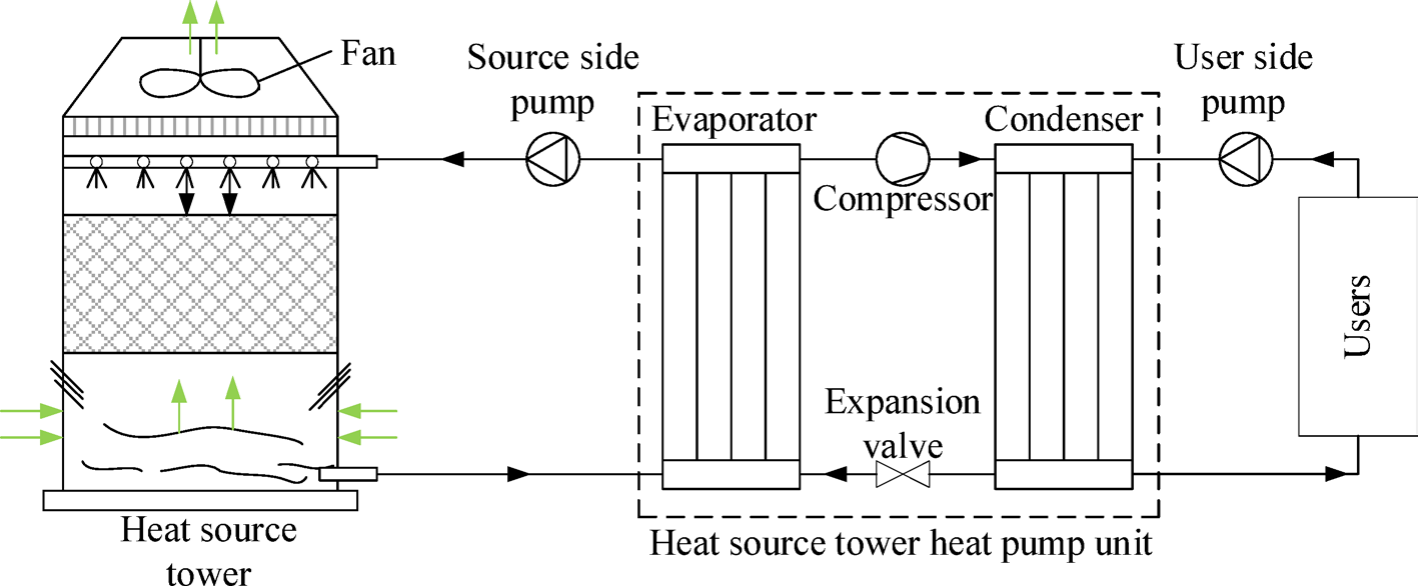
Schematic diagram of open countercurrent HST heat pump system.

The operating principle of the open HST heat pump system is shown in Fig. 1, where the black arrow indicates the direction of flow of the solution and the light green arrow indicates the direction of flow of air. In winter heating conditions, antifreeze solution is sprayed by the nozzle to the packing, and the bottom of the tower countercurrent upward air contact; the sensible and latent heat in the air is absorbed by the solution so that the solution temperature rises. The heated solution enters the evaporator side of the heat pump unit in the HST and transfers the heat to the low-temperature refrigerant in the evaporator of the heat pump unit to evaporate into a low-temperature, low-pressure gas, while the solution itself is cooled and returned to the top of the tower to circulate the spray. The evaporated refrigerant is pressurized and warmed up by the compressor and then enters the indoor side condenser to release heat and heat the indoor circulating water or air to realize heating. The refrigerant is condensed into a liquid state and then returns to the evaporator through the throttle valve to complete the cycle. It is worth noting that the refrigerant used in the heat pump system is the high GWP refrigerant R134a, which is being gradually phased out globally, so the use of low GWP refrigerants such as HFOs and natural refrigerants will become the direction of future industry development.

The HST heat pump system consists of an open countercurrent HST, an HST heat pump units, and a tower-side solution circulating pump. The detailed parameters of the equipment utilized in the experiment are outlined in Table 1.

**Table 1:** Main test equipment.

Instrument	Type	Measuring range	Accuracy
Clamp power meter	PM2203	0.1–600 kW	± 3.0%
Ultrasonic flowmeter	TUF-2000P	0–32 m/s	± 1.0%
Portable heat meter	TUF-2000H	0–10 m/s − 30–160 °C	± 1.0% ± 1.0%
Hot-wire anemometer	F925	0.40–25.00 m/s	± 2%
Temperature and humidity meter	JR900	Temperature: -10–50 °C Relative humidity: 0.0%-100%	Temperature: ± 1 °C Relative humidity: ± 5%

### Establishment of mathematical model

**Fig. 2:**
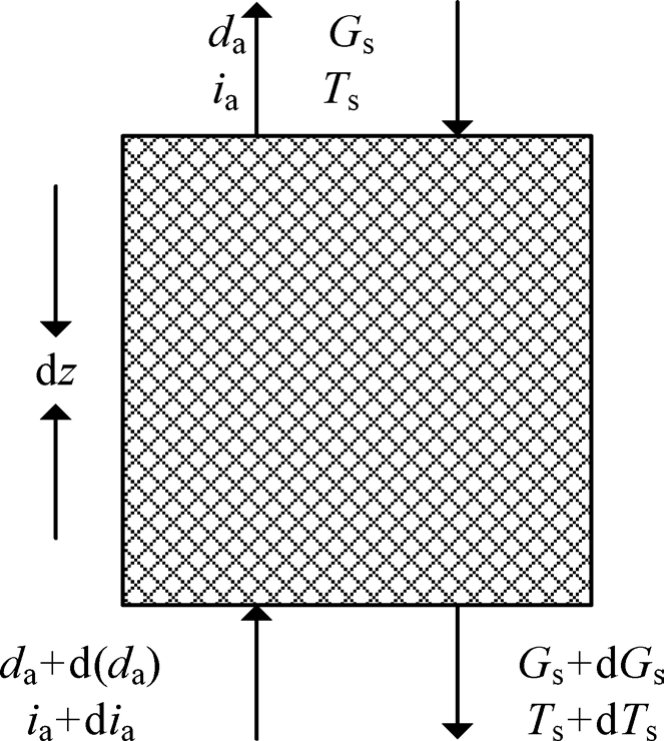
An infinitesimal control volume in an open countercurrent HST.

The system analysis by selecting a small control element within the packing is presented in Fig. 2. The primary assumptions and simplifications made for the mathematical model are as follows:

1. Heat and mass transfer are considered only along the height direction of the packing material; the radial influence on heat and mass transfer within the packing is neglected.
2. The packing properties are uniform and stable, and the heat and mass transfer coefficients remain consistent within the packing.
3. The solution is always in thermal equilibrium with the air during thermal mass exchange.
4. The heat and mass transfer area on the packing surface is the same.

According to the conservation of mass, the mass exchange equilibrium between the solution and the moist air in the column can be expressed as^29^:

1 $${\text{d}}G_{{\text{s}}} = G_{{\text{a}}} {\text{d}}\left( {d_{{\text{a}}} } \right)$$

where *G*_s_ and *G*_a_ are the flow rates of solution and air, respectively, kg/s; *d*_a_ is air humidity ratio, kg/kg.

Based on the conservation of energy, the enthalpy changes due to the concentration change during the heat and mass exchange process within the selected control volume element are negligible. Consequently, the total amount of heat exchange of air can be expressed as follows:

2 $$c_{{\text{s}}} G_{{\text{s}}} {\text{d}}T_{{\text{s}}} + c_{{\text{s}}} T_{{\text{s}}} {\text{d}}G_{{\text{s}}} = G_{{\text{a}}} i_{{\text{a}}}$$

where *c*_s_ is the specific heat capacity of the solution, kJ/(kg·°C); *T*_s_ is the solution temperature, °C; *i*_a_ is the enthalpy of air, kJ/kg.

Furthermore, wet air enthalpy can be calculated from the following expression:

3 $$i_{{\text{a}}} = c_{{\text{p,a}}} T_{{\text{a}}} + {\text{d}}_{{\text{a}}} \left( {r_{{0^{ + } }} c_{{\text{p,v}}} T_{{\text{a}}} } \right)$$

where *c*_p,a_ and *c*_p,v_ are the constant pressure specific heat of dry air and water vapor, respectively; *r*_0_ is the vaporization heat of water at 0 °C; *T*_a_ is the air temperature. In this study, *c*_p,a_ = 1005 kJ/(kg °C), *c*_p,v_ = 1842 kJ/(kg·°C), and *r*_0_ = 2500 kJ/kg.

The differential form of Eq. (3) can be expressed in the form below:

4 $${\text{d}}i_{{\text{a}}} = \left( {c_{{\text{p,a}}} + c_{{\text{p,v}}} d_{{\text{a}}} } \right)T_{{\text{a}}} + \left( {r_{0} + c_{{\text{p,v}}} T_{{\text{a}}} } \right){\text{d}}i_{{\text{a}}}$$

The mass transfer of the control volume is equal to the mass change of the olution. This can be mathematically expressed as follows:

5 $${\text{d}}G_{{\text{s}}} = ah{\text{m}}A\left( {d_{{\text{s}}} - d_{{\text{a}}} } \right){\text{d}}z$$

where *h*_m_ is the mass transfer coefficient driven by moisture content, kg/(m^2^ s); *a* is the specific surface area of the packing material, m^2^/m^3^;* A* is the cross sectional area of the packing, m^2^; *d*s is the saturated air humidity ratio at the interface between solution and air, g/kg.

The combination of Eqs. (1) and (5) yields the following expression:

6 $$G{\text{ad}}\left( {d_{{\text{a}}} } \right) = ah{\text{m}}A\left( {d{\text{s}} - d{\text{a}}} \right){\text{d}}z$$

The total energy of heat and mass exchange in the control volume is:

7 $$G_{{\text{a}}} \,{\text{ d}}i_{{\text{a}}} = [i_{{\text{v}}} \,h_{{\text{m}}} (d_{{\text{s}}} - d_{{\text{a}}} ) + h_{{\text{c}}} (T_{{\text{s}}} - T_{{\text{a}}} )]aA\,{\text{d}}z$$

8 $$i_{{\text{v}}} = r_{{0}} + c_{{\text{p,v}}} \,T_{{\text{v}}}$$

where *i*_v_ is the specific enthalpy of water vapor, J/kg; *T*_v_ represents the water vapor temperature at the gas–liquid interface, °C; *h*_c_ is the heat transfer coefficient, kW/(m^2^ °C).

The Lewis factor is defined as follows30:

9 $$L{\text{e}}_{{\text{f}}} = \frac{{h_{{\text{c}}} }}{{h_{{\text{m}}} \,c_{{\text{p,a}}} }}{ = 0}{\text{.865}}^{{2/3}} \,\frac{{\frac{{d_{{\text{s}}} + 0.622}}{{d_{{\text{a}}} + 0.622}} - 1}}{{\ln \frac{{d_{{\text{s}}} + 0.622}}{{d_{{\text{a}}} + 0.622}}}}$$

The combination of Eqs. (4), (6), (7), (8), and (9) yields the following expression:

10 $$G_{{\text{a}}} \left( {c_{{{\text{p}},{\text{a}}}} + c_{{{\text{p}},{\text{v}}}} d_{{\text{a}}} } \right){\text{d}}T_{{\text{a}}} = \left( {1 - r_{{0}} - c_{{\text{p,v}}} T_{{\text{a}}} } \right)[h_{{\text{m}}} \left( {d_{{\text{s}}} - d_{{\text{a}}} } \right)){(}r_{{0}} + c_{{\text{p,v}}} T_{{\text{v}}} {) + }h_{{\text{c}}} \,Le_{{\text{f}}} {(}T_{{\text{s}}} - T_{{\text{a}}} {)]}aA\,{\text{d}}z$$

Ignoring the small terms, Eq. (10) can be simplified in the form below:

11 $$G_{{\text{a}}} \left( {c_{{\text{p,a}}} + c_{{\text{p,v}}} \,d_{{\text{a}}} } \right){\text{d}}T_{{\text{a}}} = ah_{{\text{m}}} \,c_{{\text{p,a}}} \,{(}T_{{\text{a}}} - T_{{\text{A}}} {\text{)d}}z$$

Introducing Eqs. (2), (5), (7), (8), and (9) in Eq. (11) results in the following expression:

12 $$G_{{\text{s}}} {\text{d}}T_{s} = \left[ {h_{{\text{m}}} \left( {r_{0} + c_{{\text{p,v}}} } \right)\left( {d_{{\text{s}}} - d_{{\text{a}}} } \right) + aAh_{{\text{c}}} \,Le_{{\text{f}}} \left( {T_{{\text{s}}} - T_{{\text{a}}} } \right) - c_{s} T_{s} \left[ {ahA\left( {d_{{\text{s}}} - d_{{\text{a}}} } \right)} \right.} \right]{\text{d}}z$$

Similarly, Eq. (12) can be simplified in the form below:

13 $$c_{{\text{s}}} G_{{\text{s}}} {\text{d}}T_{{\text{s}}} = ah_{{\text{m}}} A\left[ {\left( {c_{{\text{p,a}}} \,Le_{{\text{f}}} {(}T_{{\text{s}}} - T_{{\text{a}}} {)}} \right.{ + (}d_{{\text{s}}} - d_{{\text{a}}} {)}\left( {r_{{0}} + c_{{\text{p,v}}} T_{{\text{a}}} { - }c_{{\text{s}}} T_{{\text{s}}} } \right)} \right]{\text{d}}z$$

Equations (5), (6), (11), and (13) represent the differential equations governing the heat and mass transfer of the countercurrent open HST. Four target variables need to be solved: the solution outlet temperature, air outlet dry bulb temperature, air outlet humidity ratio, and solution flow rate.

### Auxiliary equations

The convective heat transfer coefficient *h*_c_ and the convective mass transfer coefficient *h*_m_ can be calculated using the following expressions (26):

14 $$h_{c} = \frac{{k\left( {2.0 + 0.6\,{\text{Re}}^{0.5} \,\Pr^{0.33} } \right)}}{H}$$

15 $$h_{m} {\text{m}} = \frac{{D\left( {{2 + 0}{\text{.6}}\,{\text{Re}}^{{{0}{\text{.5}}}} \,Sc^{0.33} } \right)}}{H}$$

where* k* = 0.0244 W/(m·°C) is the thermal conductivity of wet air; *Re* is the Reynolds number; *Pr* is the Planck constant; *H* denotes the packing spacing, m; *D* is the diffusion coefficient of water vapor in the air, m^2^/s; *Sc* represents the Schmidt number.

The diffusion coefficient is defined as follows^31^:

16 $$D = \frac{{{435}{\text{.7}}\left( {T_{{\text{a}}} + {273}{\text{.15}}} \right)\left( {\frac{{1}}{{M_{{\text{A}}} }} + \frac{{1}}{{M_{{\text{B}}} }}} \right)^{{\frac{{1}}{{2}}}} }}{{P_{{0}} \left( {V_{{\text{A}}}^{{\frac{{1}}{{3}}}} + V_{{\text{B}}}^{{\frac{{1}}{{3}}}} } \right)^{{2}} }} \times {10}^{{{ - }{4}}}$$

where *P*_0_ = 101325 Pa is the atmospheric pressure; *M*_A_ = 18 g/mol and *M*_B_ = 28.9 g/mol are the molar mass of water vapor and air, respectively; *V*_A_ =18.9 cm^3^/(g·mol) and *V*_B_ = 29.9 cm^3^/(g·mol) are the molar volume of water vapor and air, respectively.

According to the empirical equation derived by Conde et al.^32^, the specific heat of CaCl_2_ at constant pressure can be obtained using the following expression:

17 $$c_{{\text{s}}} = c_{{{\text{pH}}_{{2}} {\text{o}}}} { [1} - f_{{1}} {(}T_{{\text{a}}} {)}f_{{2}} {(}\varepsilon {)]}$$

where *ε* is the mass concentration of the solution.

### Numerical solution

**Fig. 3:**
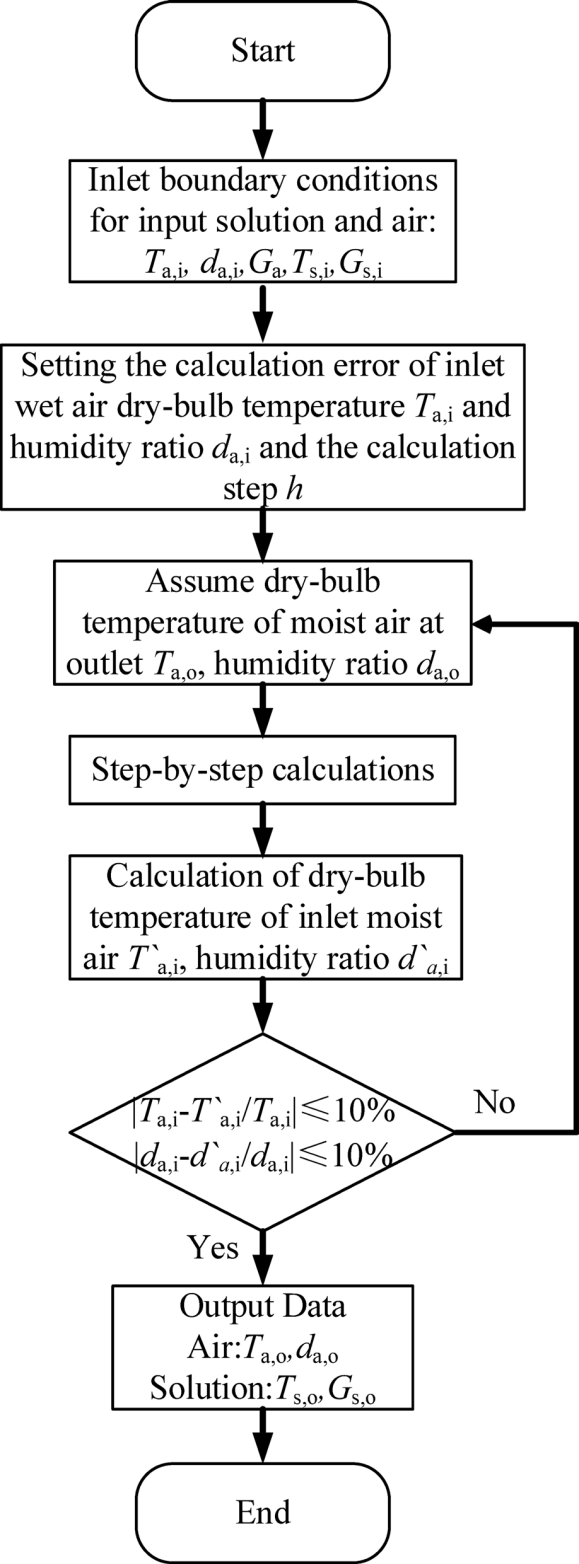
Calculation flowchart.

Following the method employed by Kasim et al.^33^ for solving fourth-order ordinary differential equations, the equations are solved using the traditional fourth-order Runge–Kutta method. Since the ordinary differential equations in this paper involve four variables, solving them individually with the Runge–Kutta method can be complex. To simplify calculations, the multivariable is regarded as a single variable, and the transformation is as follows:

18 $$K_{{1}} = f{(}told_{{\text{n}}} ,\,uold_{{\text{n}}} {)}$$

19 $$K_{{2}} = f\left( {told_{{\text{n}}} + \frac{h}{{2}},\,uold_{{\text{n}}} + \frac{h}{{2}}K_{{1}} } \right)$$

20 $$K_{{3}} = f\left( {told_{{\text{n}}} + \frac{h}{{2}},uold_{{\text{n}}} { + }\frac{h}{{2}}K_{{2}} } \right)$$

21 $$K_{{4}} = f{(}told_{{\text{n}}} + h,uold_{{\text{n}}} + hK_{{3}} {)}$$

22 $$uold_{{n + {1}}} = uold_{{\text{n}}} + \frac{h}{{6}}{(}K_{{1}} + {2}K_{{2}} + {2}K_{{3}} + K_{{4}} {)}$$

where *told* is the time from the previous step, and *uold* is a variable that deforms multiple variables. The iterative calculations are performed using MATLAB software, proceeding layer by layer down the height of the HST packing to derive the performance prediction results of the HST. The calculation flowchart is presented in Fig. 3.

### Model validation

**Fig. 4:**
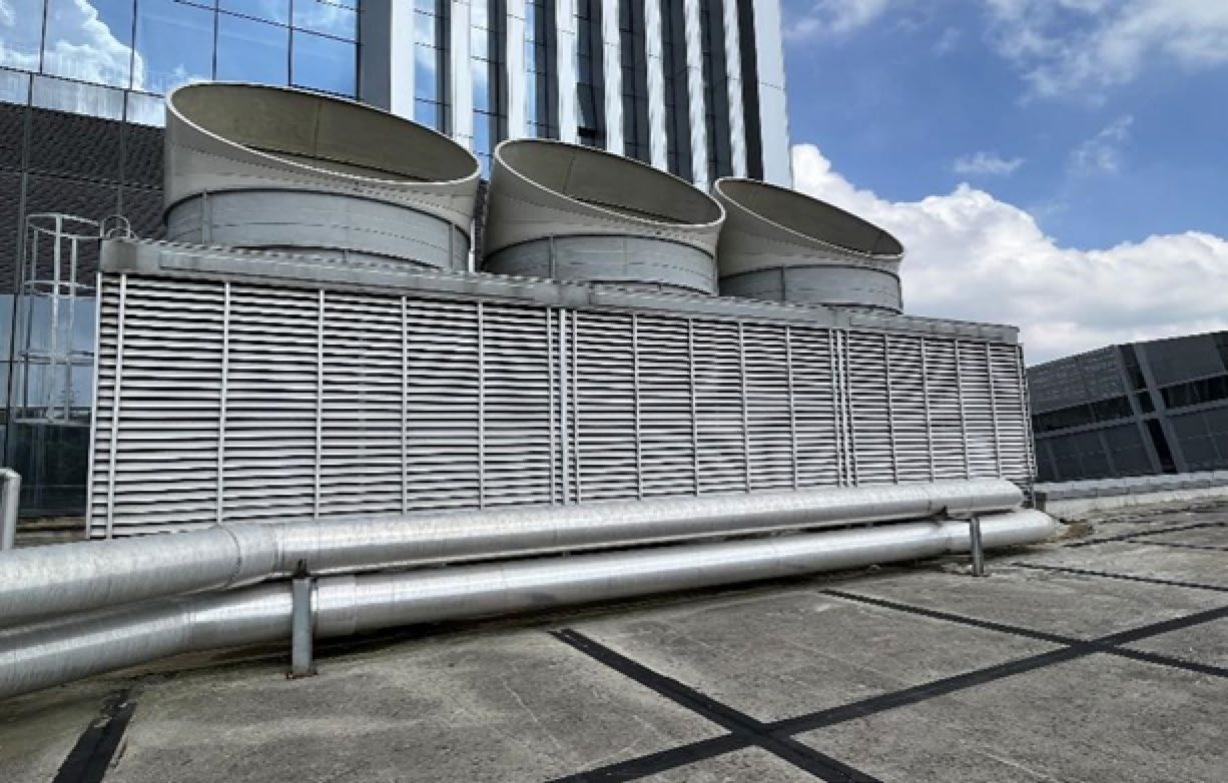
The studied countercurrent HST.

To validate the accuracy of the established heat and mass transfer model, a field test was conducted on the countercurrent open HST heat pump system in a building located in Guizhou Province, China, as depicted in Fig. 4. It is worth noting that Guizhou Province has a warm and humid climate, falling under the subtropical warm and humid monsoon climate zone. The region receives abundant precipitation, with hot and rainy conditions prevailing in the same season, leading to an annual relative humidity exceeding 70%. Due to atmospheric circulation patterns and topography, Guizhou’s climate exhibits diversity. The HST dimensions are 4200 mm in length, 6300 mm in width, and 5400 mm in height. The packing consists of corrugated plates with a specific surface area of 300 m^2^/m^3^. The circulating medium employed in the system is a CaCl_2_ solution.

**Fig. 5:**
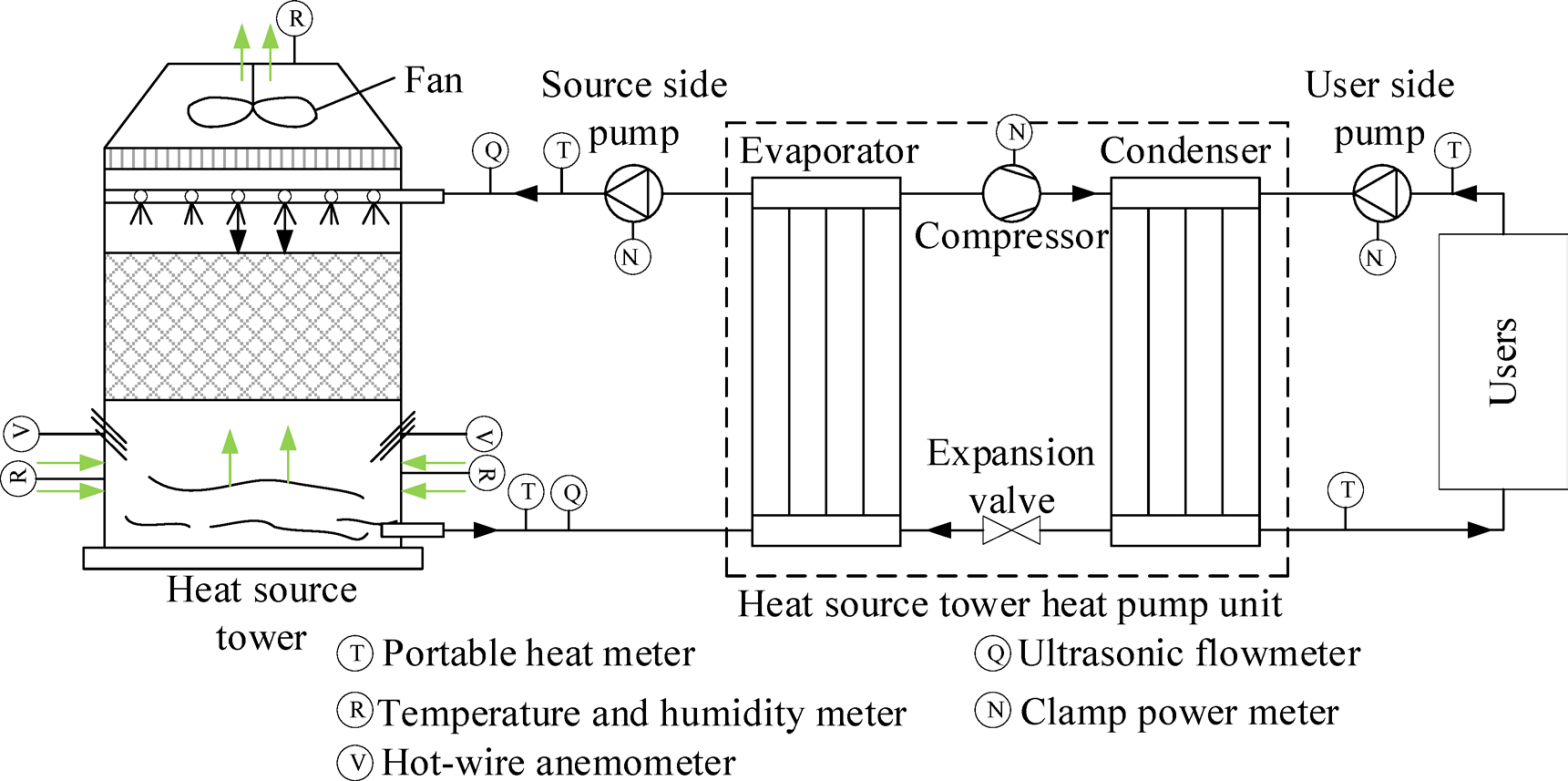
Instrumentation measurement points layout.

In the field tests, the parameters were read and recorded at 10-min intervals using the instrumentation in Table 1 of Section “Working principle of HST”, for a duration of 9 h per day. The main key parameters tested are solution temperature, solution flow rate, hot water temperature, air temperature, air relative humidity, inlet air velocity and power of the equipment. Before each test, the instrument is calibrated to ensure high accuracy. In addition, the tests were conducted after the parameters of the heat pump system in the HST had stabilized in operation. To obtain the air mass flow rate, the cross-section of the air inlet was divided into 15 uniformly sized sections, and the air velocity was measured at the center of the sections and calculated from the arithmetic mean of the air velocity and the cross-sectional area. In order to the accuracy of solution flow and temperature testing, ultrasonic flowmeters and portable heat meters were used, and the test points were selected according to the testing principles, and the arrangement of the test points is shown in Fig. 5. It is important to note that the solution concentration unit was not in operational use during the testing of this study and therefore its power was not tested. The test values for the main test parameters can be found online in Supplementary Table S1.

The model incorporates the relevant parameters of the inlet air and solution obtained through testing as known conditions to calculate the outlet air and solution temperatures. Simultaneously, the root mean square error (*RMSD*) is introduced for error analysis:

23 $$RMSD = \sqrt {\frac{{\sum {{[(}X_{{\text{a,o}}} - X_{{\text{a,i}}} {)}/X_{{\text{a,i}}} {]}^{2} } }}{N}}$$

where *X*_a,o_ and *X*_a,i_ are the measured and calculated values, respectively; *N* is the total number of datasets.

**Fig. 6:**
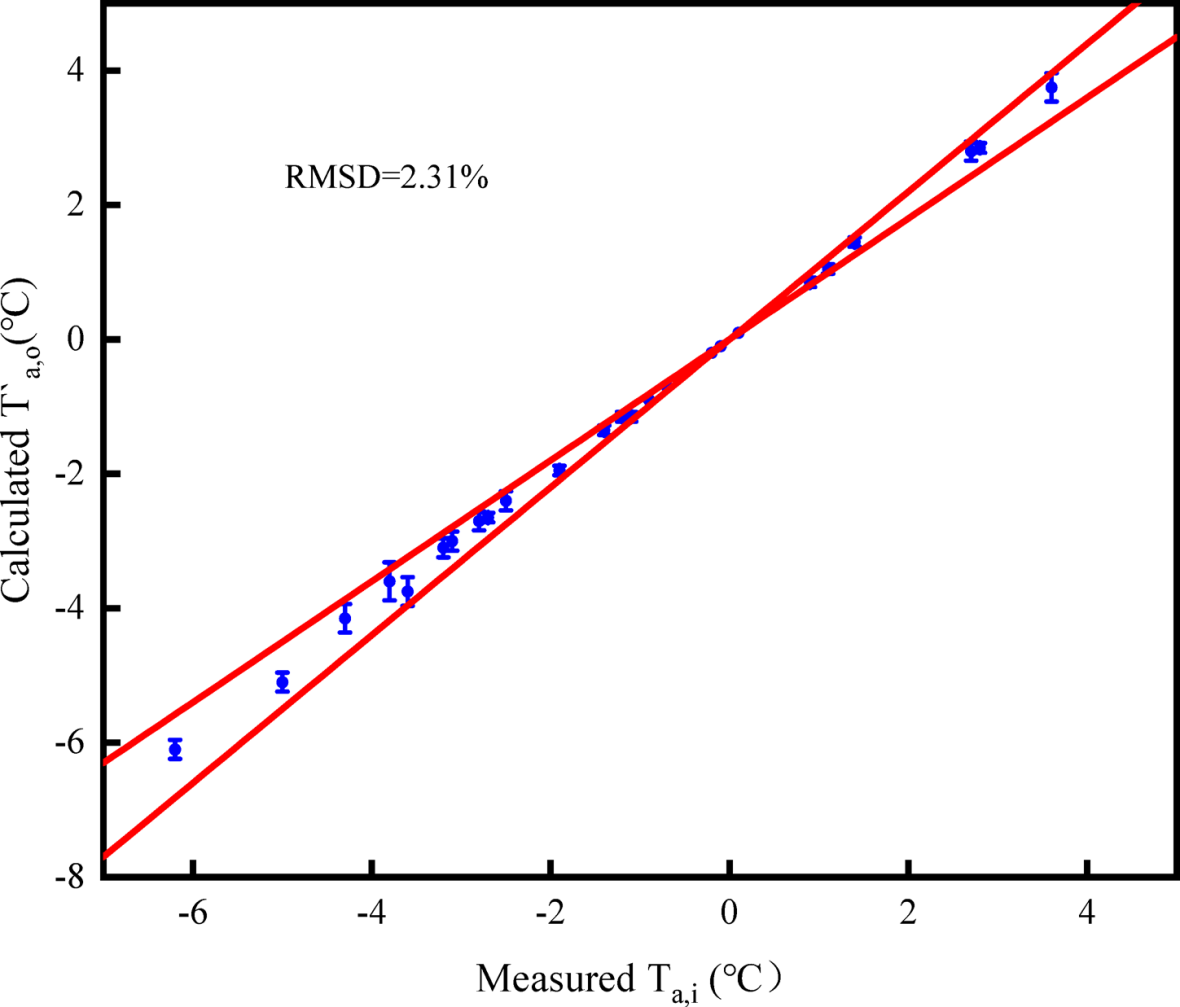
Comparison of calculated and measured values of outlet air temperature.

**Fig. 7:**
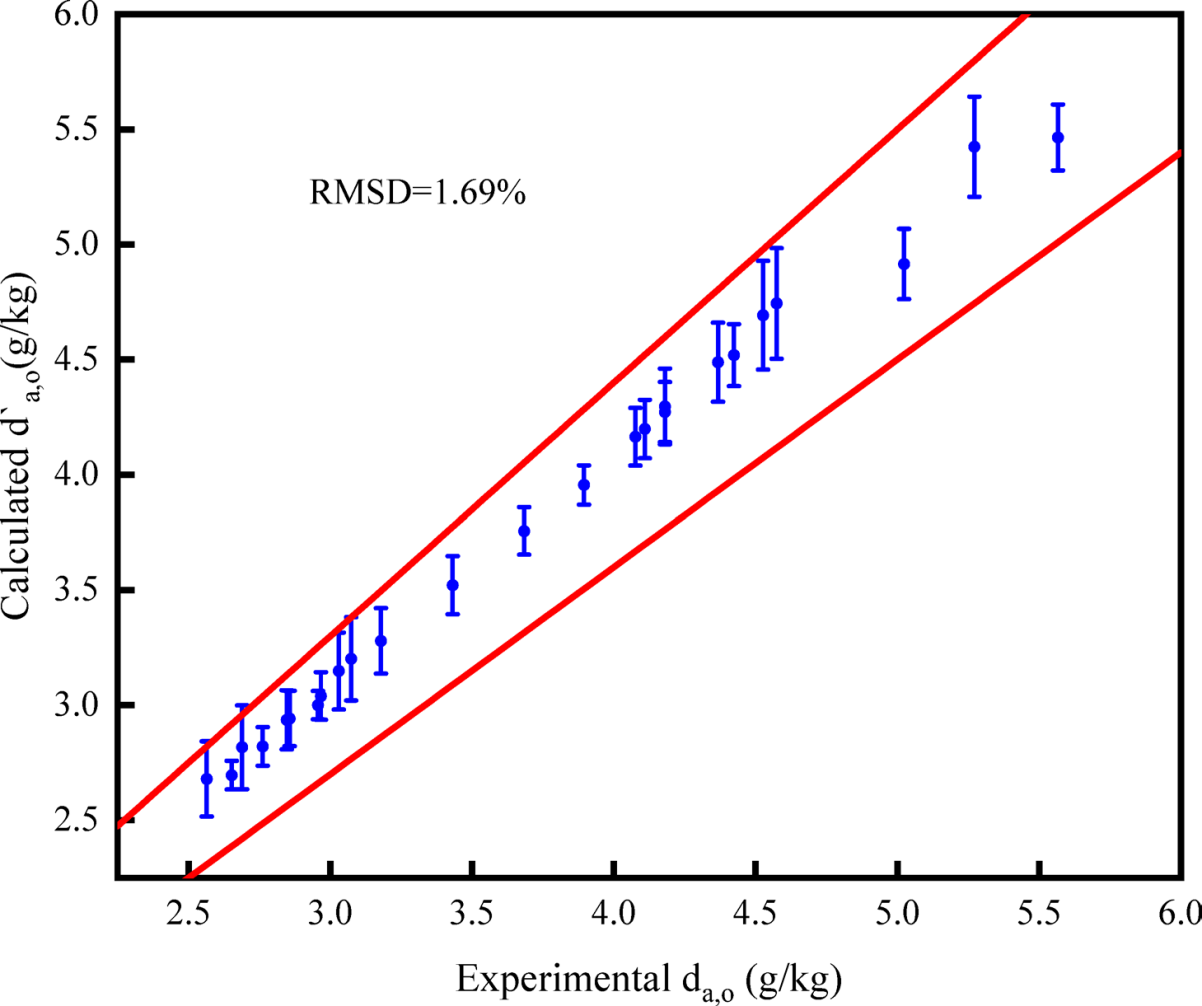
Comparison of calculated and measured values of outlet air moisture content.

**Fig. 8:**
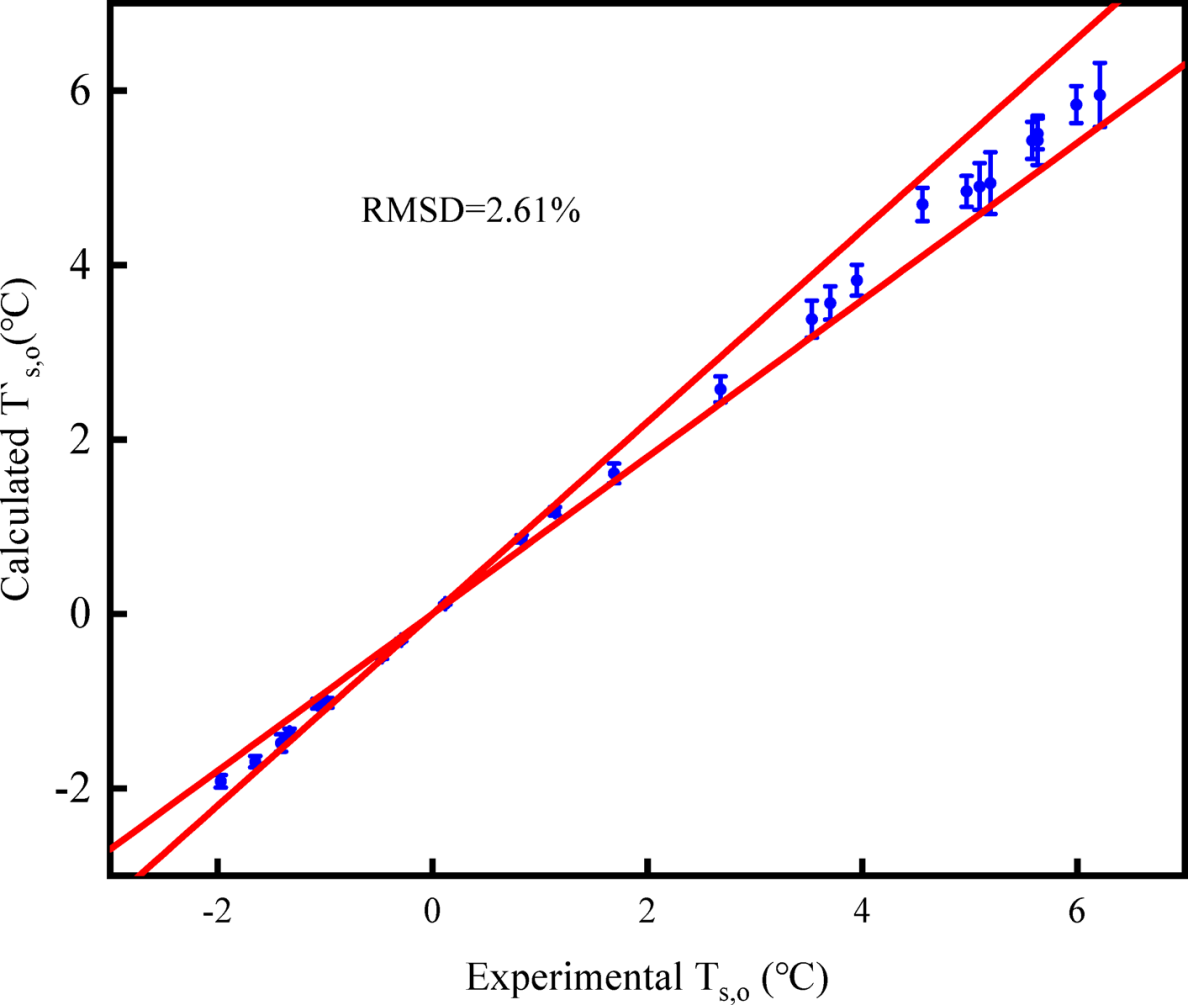
Comparison of calculated and measured values of outlet solution temperature.

Figures 6, 7, and 8 illustrate the errors between the measured values of outlet air temperature, outlet air humidity ratio, and outlet solution temperature, and the calculated values of the model. According to Eq. (23), the *RMSD* values of outlet air temperature, outlet air humidity ratio, and solution temperature are 2.31%, 1.69%, and 2.61%, respectively. It is found that the error of the three parameters is within 5%. The model accurately predicts the heat and mass transfer performance of the open countercurrent HST.

## Analysis of influencing factors of heat and mass transfer in HST

### Heat and mass transfer performance indicators and working conditions

In order to study the heat transfer effect of the countercurrent HST in the low temperature and high humidity region, the heat transfer performance of the HST is characterized by the total heat transfer, sensible heat transfer, latent heat transfer, and latent heat ratio; at the same time, according to the established model, a single-factor analysis is used to change the inlet air temperature, the inlet solution temperature, and the relative humidity to study the effect of air and solution parameters on the heat transfer of the tower (see Table 2). In addition, several sets of test values and calculated values for the total heat transfer of HSTs are compared in Supplementary Table S2, from which it can be concluded that the model can be used to predict the thermal performance of HSTs.

24 $$Q_{{\text{z}}} = Q_{{\text{X}}} + Q_{{\text{q}}}$$

25 $$Q_{{\text{x}}} = {(}C_{{{\text{pa}}}} + w_{{\text{a}}} \times C_{{{\text{pv}}}} {)(}T_{{\text{a,o}}} - T_{{\text{a,i}}} {)}$$

26 $$Q_{{\text{q}}} = r \times G_{{\text{a}}} \left( {w_{{\text{a,o}}} - w_{{\text{a,i}}} } \right)$$

27 $$\eta = Q_{{\text{q}}} /Q_{{\text{z}}}$$

where *Q*_z_ is the total heat transfer, kW; *Q*_x_ is the sensible heat transfer, kW; *Q*_q_ is the latent heat transfer, kW; *η* is the latent heat ratio.

**Table 2:** Simulation conditions in open countercurrent HST.

Parameter	Inlet air temperature (°C)	Inlet solution temperature (°C)	Relative humidity (%)	Inlet air mass flow rate (kg/s)	Inlet solution mass flow rate (kg/s)
Inlet air temperature	− 3~5	− 2	88	50	87.5
Inlet solution temperature	5	− 4~4	88	50	87.5
Relative humidity	5	− 2	60~100	50	87.5

### Influence of inlet air temperature and relative humidity on heat and mass transfer

**Fig. 9:**
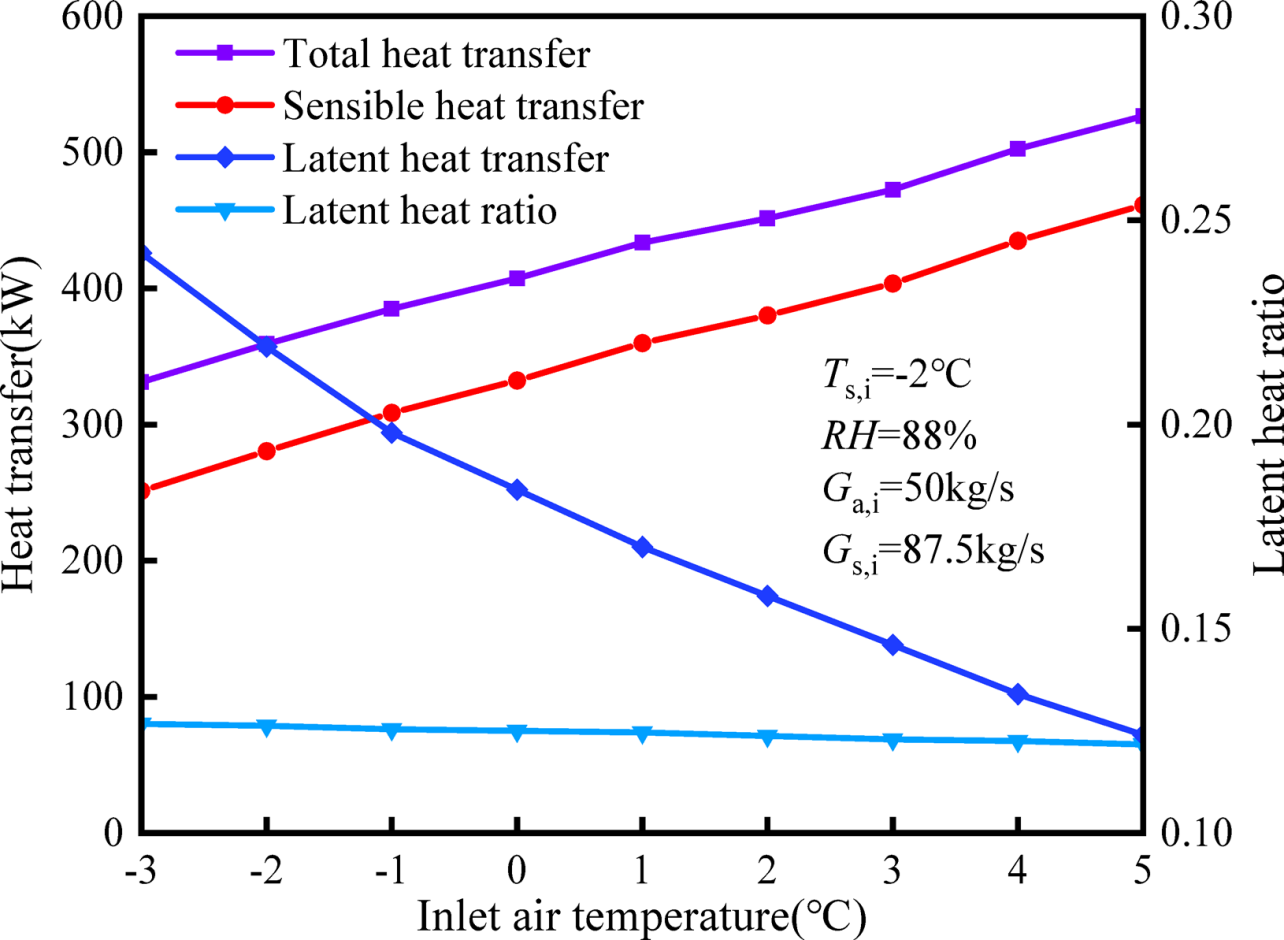
Influence of inlet air temperature on heat and mass transfer.

Figure 9 illustrates the heat exchange of the HST under various inlet air temperatures. It is observed that as the inlet air temperature increases, the corresponding sensible heat exchange rises. More specifically, when the inlet air temperature increases from − 3 to 5 °C, sensible heat exchange increases from 251.25 to 461.27 kW, representing a notable 83.59% rise in sensible heat exchange. Conversely, there is a slight decrease of 18.75% in latent heat exchange, and the proportion of latent heat exchange to total heat exchange gradually diminishes at a decreasing rate. This phenomenon may be attributed to the rise in air temperature, which raises the solution temperature in the tower. Consequently, sensible heat exchange increases due to the enhanced heat exchange capacity resulting from the temperature increase. This temperature increase also reduces the transfer of water vapor from the air to the solution, causing a decline in latent heat exchange. Figure 9 indicates that the impact of air temperature on latent heat transmission is considerably less pronounced compared to sensible heat exchange.

**Fig. 10:**
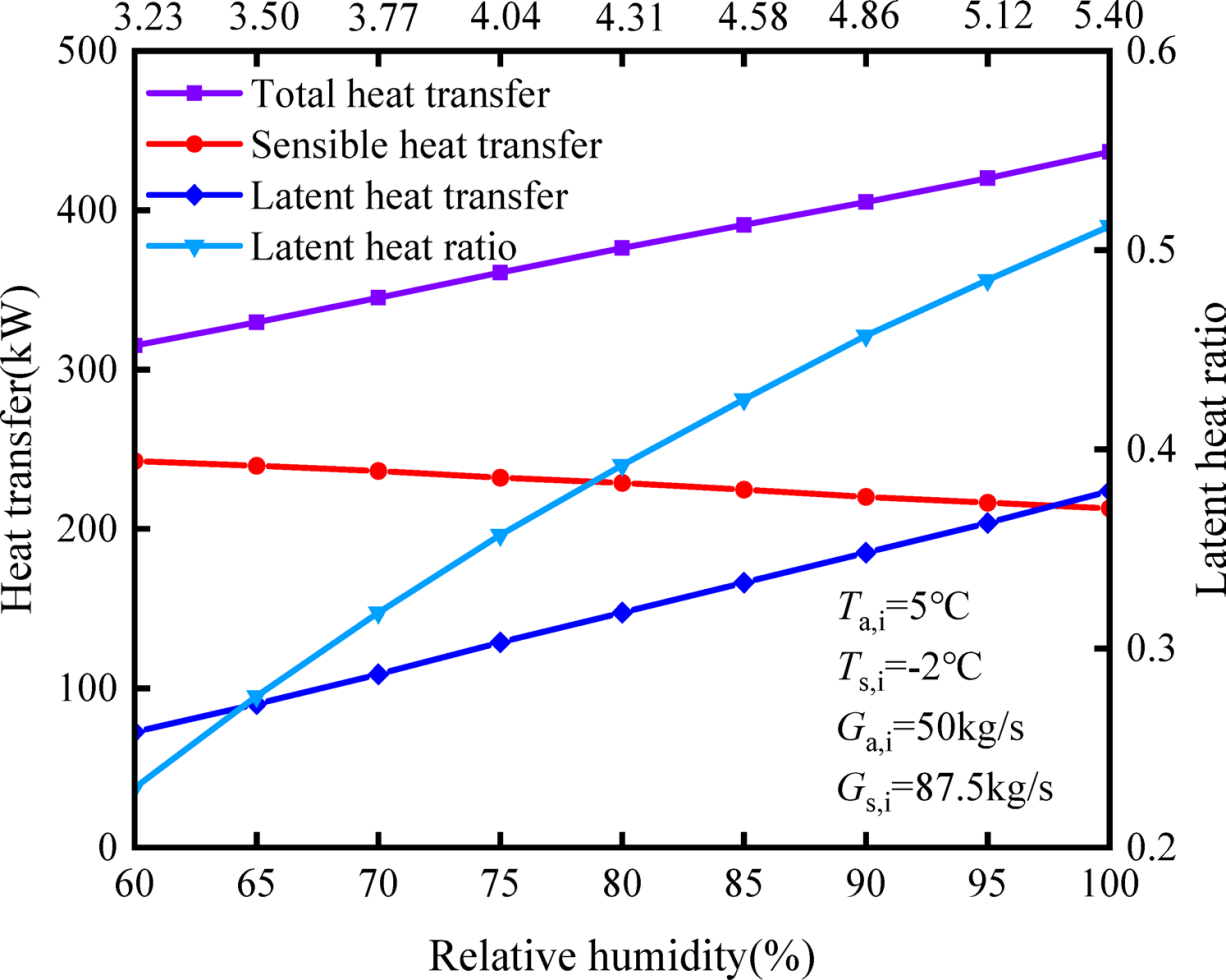
Influence of relative humidity on heat and mass transfer.

Figure 10 illustrates the impact of various relative humidities on the heat exchange of the HST. It is observed that as the relative humidity increases, latent heat exchange increases. Specifically, as the latent heat exchange increases from 72.5 to 223.75 kW, the corresponding latent heat ratio increases from 0.23 to 0.512. This elevation in latent heat exchange originates from the increase in water vapor partial pressure in the incoming air as relative humidity rises. Consequently, there is a greater difference in water vapor partial pressure between the air and the solution, facilitating the transfer of water vapor from the humid air to the solution. Meanwhile, the results demonstrate that as the relative humidity rises, the corresponding sensible heat transmission reduces slightly, decreasing from 242.58 to 212.96 kW. However, the overall heat exchange increases, primarily driven by the enhanced latent heat exchange. Therefore, during operation in high humidity conditions, the HST faces a risk of freezing and heightened energy consumption for regeneration, given the instability of the freezing point of the antifreeze solution in such circumstances.

### Influence of inlet solution temperature on heat and mass transfer

**Fig. 11:**
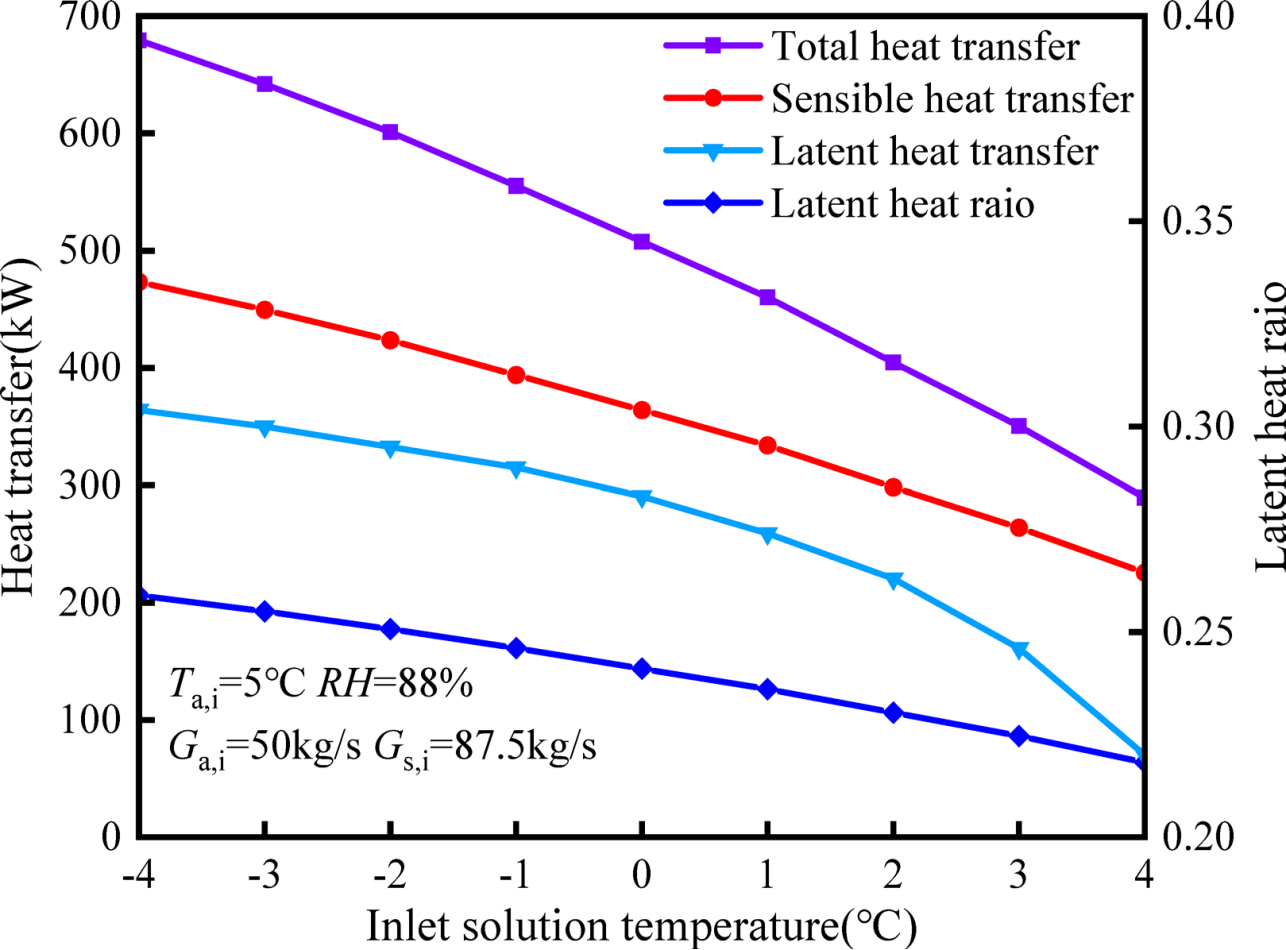
Influence of inlet solution temperature on heat and mass transfer.

Figure 11 illustrates the impact of different inlet solution temperatures on heat exchange. It is observed that as the inlet solution temperature rises, both sensible heat exchange and latent heat exchange decrease. Specifically, there is a 52.3% decrease in sensible heat exchange, a 69.1% decrease in latent heat exchange, and a 57.4% decrease in total heat exchange. Furthermore, as the solution temperature increases, the rate at which the latent heat ratio decreases becomes more pronounced. The concurrent reduction in sensible heat and latent heat may be attributed to the effect of the solution temperature on heat and mass exchange within the tower. The diminishing temperature difference between the solution and the air, as well as the decreasing water vapor partial pressure differential between the humid air and the solution in the tower, originate from the solution temperature increase. These results demonstrate the substantial impact of the solution temperature on heat and mass exchange. Raising the temperature of the incoming solution can reduce the moisture absorption of the solution, thereby mitigating the risk of freezing. Consequently, when the heating capacity is sufficient, it is feasible to raise the temperature of the incoming solution while operating the HST.

## Heating performance analysis of heat source tower heat pump system

### Performance evaluation indicators

In order to understand the operating and working characteristics of the heat tower heat pump system in low temperature and high humidity environments, the coefficient of performance (*COP*) (Eq. 28), the heating seasonal coefficient of performance (*SCOP*) (Eq. 30) and the theoretical *COP*_t_ (Eq. 29) were selected to evaluate the heating performance of the heat pump. In this article, the *COP* test period spans from January 1, 2023, to January 5, 2023, encompassing typical low-temperature and high-humidity weather in the local area. The *SCOP* test period extends from November 15, 2022, to March 25, 2023, covering an entire heating season. Table 3 presents the equipment parameters for the HST heat pump system.

28 $$COP = \frac{{{1000}c\,G_{{\text{s}}} {(}T_{{\text{s,o}}} - T_{{\text{s,i}}} {)}}}{{{3600(}E_{{\text{e}}} { + }E_{{\text{s}}} { + }E_{{\text{p}}} { + }E_{{\text{f}}} {)}}}$$

29 $$COP_{{\text{t}}} = T_{{{\text{cond}}}} /(T_{{{\text{cond}}}} - T_{{{\text{evap}}}} )$$

30 $$SCOP{ = }\frac{\sum Q }{{\sum E }}$$

where *E*_e_ is the power of the heat pump, kW; *E*_s_ and *E*_p_ denote the power of the user side and source side pump, respectively, kW; *E*_f_ is the power of the HST fan, kW; *∑Q* is the cumulative heating capacity in the heating season, kW·h; *∑E* represents the cumulative power consumption in the heating season, kW·h; *T*_cond_ is the condensation temperature, K; *T*_evap_ is the evaporation temperature, K.

**Table 3:** Heat tower heat pump system equipment parameters.

Name	Quantity	Type	Specifications
Heat source tower heat pump unit	3	11100S/L-R2(R134a)	Power: 233 kW
Open heat source tower	3	XPS-E9S	Power: 15 kW
User side pump	4	DFWH150-400B/4/30	Power: 30 kW
Source side pump	4	DFWH150-400A/4/37	Power: 37 kW

### Energy efficiency analysis of the heating season

**Fig. 12:**
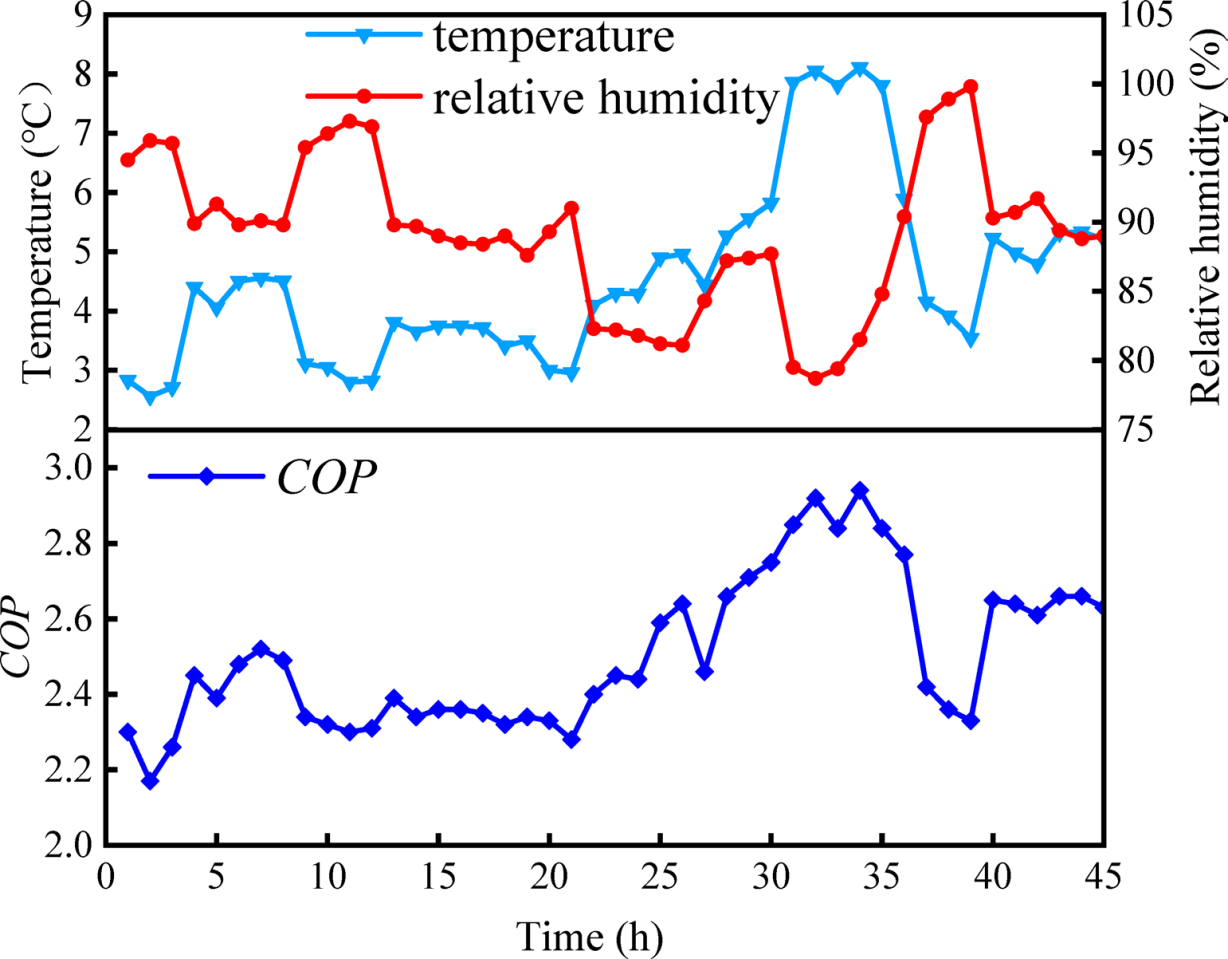
Variations of outdoor air temperature, relative humidity, and *COP* over the test period.

Figure 12 illustrates the continuous changes in temperature, humidity, and *COP* over time from January 1 to January 5. The graph reveals a positive correlation between *COP* and outdoor air temperature and a negative correlation with relative humidity. The highest *COP* value occurred at the 36th hour of the test, reaching 2.94. This peak may be attributed to the increased inlet water temperature of the evaporator and condenser, facilitated by the rising air temperature during this test period. Higher evaporator water temperature enhances the *COP* of the system. At this specific moment, the outdoor air temperature reaches its peak value, resulting in lower power consumption by the heat pump unit and water pump, thereby increasing *COP*. Conversely, the lowest *COP* value, recorded at the 2nd hour of the test, was 2.18. This low value may originate from the substantial impact of outdoor air temperature on *COP*. At this point in the test phase, the outdoor air temperature reaches its lowest value, thereby increasing the power consumption by the heat pump unit and water pump. The elevated water flow also contributes to higher power in the water pump. According to Eq. (28), an increase in the power of the heat pump unit and pump results in a decrease in *COP*. Therefore, it is essential to dynamically adjust the operating parameters of the heat pump unit based on external air parameters. Moreover, employing a frequency converter to regulate the pump may enhance the energy efficiency of the system.

**Fig. 13:**
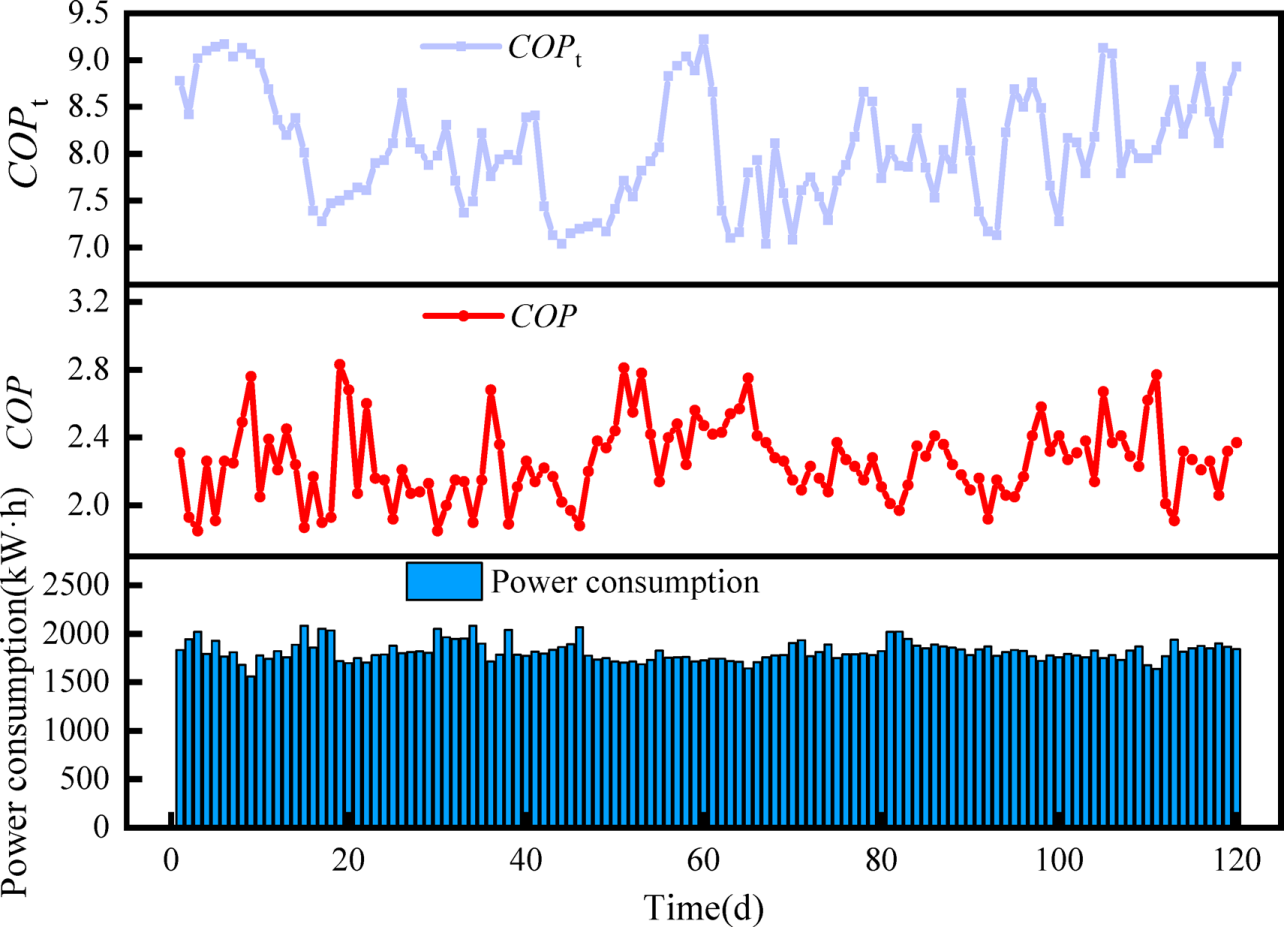
Power consumption, *COP* and *COP*_t_ over time.

Figure 13 shows the total system energy consumption, *COP* and theoretical *COP*_t_ over time throughout the heating season. It can be seen that there is a significant difference between the theoretical *COP*_t_ and the actual *COP* of the heat pump system, which is due to the fact that the theoretical *COP*_t_ is obtained based on the ideal conditions of the inverse Carnot cycle, which is mainly affected by the evaporation temperature and condensation temperature as shown in Eq. (29), while the actual *COP* is affected by the efficiency of the system components, the environmental interactions, and the operating conditions, etc. The gap between the two also shows that there is still a lot of room for improvement in the energy efficiency of heat pump systems in low-temperature and high-humidity environments. Utilizing Eq. (30), the *SCOP* is calculated to be 3.18. This value surpasses the *SCOP* limit requirement of 2.00 specified in the Chinese standard GB50736-2012 ‘Code for Design of Heating, Ventilation and Air Conditioning of Civil Buildings’^34^, The result demonstrates that the heat source tower heat pump system has advantages for winter heating in low-temperature and high-humidity areas.

## Conclusion

The present study develops a predictive model for heat and mass transfer in open countercurrent heat source towers and validates the accuracy of the model through observed data. The study explores the influence of inlet air temperature, relative humidity, and solution temperature on the heat and mass transfer efficiency of the heat source tower. Ultimately, the heating energy efficiency of the heat source tower is evaluated through an engineering scenario. The main achievements of this article can be summarized as follows:

1. As the inlet air temperature increases from − 3 to 5 °C, the sensible heat increases by 83.59%, while the latent heat decreases by 18.75%. The impact on sensible heat is more pronounced compared to latent heat.
2. As the relative humidity increases from 60 to 100%, the corresponding latent heat exchange increases from 72.5 to 223.75 kW. Meanwhile, sensible heat exchange decreases slightly, resulting in a rise in the latent heat ratio from 0.23 to 0.512. The influence of inlet air relative humidity on latent heat is more significant than its impact on sensible heat. Moreover, a high-humidity environment within the tower can lead to the transfer of water vapor from moist air to the solution, causing a dilution of the solution concentration and an increased risk of freezing and regeneration energy consumption.
3. As the inlet solution temperature increases from − 4 to 4 °C, the corresponding sensible heat and sensible heat decrease by 52.3% and 69.1%, respectively. The increase in the temperature of the solution reduces its moisture absorption, thereby mitigating the risk of freezing.
4. In typical conditions of low temperature and high humidity, the heat source tower heat pump system achieves a peak coefficient of performance of 2.94, a minimum of 2.18, and an average of 2.50. Over the entire heating season, the heat source tower heat pump system demonstrates a heating seasonal performance coefficient of 3.18, demonstrating its superiority in providing winter heating in areas characterized by low temperatures and high humidity.

## Supplementary Information

Supplementary Information.

## Data Availability

The datasets used and/or analysed during the current study available from the corresponding author on reasonable request.

## Abbreviations

HST: Heat source tower
COP: Coefficient of performance
SCOP: Heating seasonal coefficient of performance

## List of symbols

*a*: Specific surface area of packing, m^2^/m^3^
*A*: Packing cross sectional area, m^2^
*c*_s_: Specific heat capacity of a solution, kJ/(kg °C)
*c*_p,a_: Constant pressure specific heat of dry air, kJ/(kg °C)
*c*_p,v_: Constant pressure specific heat of water vapor, kJ/(kg °C)
*D*: Water vapor diffusion coefficient, m^2^/s
*d*_a_: Air humidity ratio, kg/kg
*d*_s_: Saturated air humidity ratio at the interface between solution and air, kg/kg
*E*_e_: Heat pump host power, kW
*E*_s_: Power of pump on user side, kW
*E*_p_: Source side pump power, kW
*G*_a_: Air flow rate, kg/s
*G*_s_: Solution flow rate, kg/s
*H*: Packing spacing, m
*h*_c_: Heat transfer coefficient, kW/(m^2^ °C)
*h*_m_: Mass transfer coefficient, kg/(m^2^ s)
*i*_a_: Air enthalpy , kJ/kg
*i*_v_: Specific enthalpy of water vapor,J/kg
*k*: Thermal conductivity, W/(m °C)
*M*_A_: Molar mass of water vapor, g/mol
*M*_B_: Molar mass of air, g/mol
*N*: Total number of data sets
*P*_0_: Atmospheric pressure, Pa
*Pr*: Planck number
*Re*: Reynolds number
*r*_0_: Heat of vaporization of water at 0 °C, kJ/kg
*Sc*: Schmidt number
*T*_s_: Solution temperature, °C
*T*_a_: Air temperature, °C
*T*_v_: Water vapor temperature at the gas–liquid interface, °C
*T*_evap_: Evaporating temperature, K
*T*_cond_: Condensing temperature, K
*V*_A_: Molar volume of water vapor, cm^3^/(g mol)
*V*_B_: Molar volume of air cm^3^/(g mol)
*X*_a,o_: Experimental value
*X*_a,i_: Calculated value
Z: Along the packing height direction
*∑Q*: Cumulative heat outpu, kW h
*∑E*: Cumulative power consumption, kW h

## Greek symbols

ε: Solution mass concentration

## Subscripts

a: Moist air
s: Solution
i: Inlet
o: Outlet
