# Supplementary material for: Study on the performance of open countercurrent heat source tower under low temperature and high humidity environment
Source: Sci Rep. 2025 Jul 17;15:26005. doi: 10.1038/s41598-025-09378-0 (PMC12271543; doi:10.1038/s41598-025-09378-0)
Supplement: Supplementary file 1 — Supplementary Information. [file 41598_2025_9378_MOESM1_ESM.docx]

**Supplementary Table S1**

Summary table of test values for selected parameters

| Time | *T*_a,i_(℃) | *T*_a,o_(℃) | *T*_s,i_(℃) | *T*_s,o_(℃) | *d*_a,i_(g/kg) | *d*_a,o_(g/kg) |
| --- | --- | --- | --- | --- | --- | --- |
| 9.00 | 2.61 | -3.64 | -3.622 | -0.985 | 3.49 | 2.71 |
| 9:10 | 2.94 | -3.48 | -3.746 | -1.113 | 3.44 | 2.66 |
| 9:20 | 3.26 | -3.27 | -3.612 | -0.897 | 3.47 | 2.74 |
| 9:30 | 3.21 | -3.15 | -3.287 | -0.856 | 3.46 | 2.82 |
| 9:40 | 3.15 | -3.22 | -3.553 | -1.075 | 3.44 | 2.75 |
| 9:50 | 3.24 | -3.10 | -3.734 | -1.240 | 3.39 | 2.62 |
| 10:00 | 3.36 | -3.25 | -3.321 | -0.912 | 3.41 | 2.72 |
| 10:10 | 3.13 | -3.34 | -3.703 | -0.764 | 3.52 | 2.68 |
| 10:20 | 3.17 | -3.12 | -3.569 | -0.931 | 3.48 | 2.61 |
| 10:30 | 3.04 | -3.30 | -3.634 | -1.245 | 3.47 | 2.66 |
| 10:40 | 3.10 | -3.45 | -3.786 | -1.421 | 3.51 | 2.80 |
| 10:50 | 2.92 | -3.58 | -3.845 | -1.176 | 3.45 | 2.77 |
| 11:00 | 2.95 | -3.49 | -3.569 | -1.365 | 3.41 | 2.72 |
| 11:10 | 2.99 | -3.43 | -3.692 | -1.058 | 3.46 | 2.83 |
| 11:20 | 3.12 | -3.35 | -3.893 | -1.265 | 3.44 | 2.76 |
| 11:30 | 3.24 | -3.39 | -3.742 | -1.435 | 3.56 | 2.88 |
| 11:40 | 3.37 | -3.28 | -3.525 | -1.127 | 3.54 | 2.81 |
| 11:50 | 3.43 | -3.31 | -3.703 | -0.784 | 3.57 | 2.84 |
| 12:00 | 3.51 | -3.17 | -3.907 | -0.925 | 3.52 | 2.75 |

**Supplementary Table S2**

Comparison of heat exchange between test values and calculated values for HSTs.

| *T*_a,i_  (℃) | *T*_s,i_  (℃) | *G*_s,i_  (kg/s) | *G*_a,i_  (kg/s) | Experimental heat transfer(kW) | Calculated heat transfer(kW) |
| --- | --- | --- | --- | --- | --- |
| 3.17 | -3.569 | 76.7 | 52.5 | 452 | 487 |
| 2.61 | -3.622 | 79.4 | 54.8 | 435 | 468 |
| 2.92 | -3.845 | 77.5 | 55.1 | 447 | 483 |
| 3.36 | -3.321 | 78.2 | 54.9 | 467 | 498 |
| 3.51 | -3.907 | 79.8 | 55.3 | 481 | 535 |
